# Barriers and Facilitators of COVID-19 Vaccination Outreach Program in Rural India: A Qualitative Study

**DOI:** 10.7759/cureus.35800

**Published:** 2023-03-05

**Authors:** Umesh Kawalkar, Vijay Balkar, Dinesh Naitam, Sanjeev Choudhari, Manish Sharma, Hari Pawar, Manoj S Patil

**Affiliations:** 1 Community Medicine, Government Medical College (GMC) Akola, Akola, IND; 2 Dentistry, Government Medical College (GMC) Akola, Akola, IND; 3 District Reproductive and Child Health, Public Health, Zilla Parishad, Akola, IND; 4 Public Health, Government of Maharashtra, Akola, IND; 5 Research and Development, Jawaharlal Nehru Medical College, Datta Meghe Institute of Higher Education and Research, Wardha, IND

**Keywords:** india, vaccination, outreach, barriers, facilitators, covid-19

## Abstract

Background

Primary health centres are in charge of effectively implementing the COVID-19 vaccination program in rural areas. So, the study was planned to seek insight into the challenges faced by health personnel in the effective implementation of the COVID-19 vaccination program.

Methodology

The study was conducted in a rural area of Akola district which lies in the western parts of Maharashtra State and belongs to the Vidarbha region and is said to be one of the progressive districts in the region. A qualitative study was planned to understand the barriers and facilitators of the COVID-19 vaccine implementation program at rural and tribal areas. The study participants were medical officers from rural and tribal areas who actively planned and implemented COVID-19 vaccination at the primary health centre. A total of 30 medical officers were interviewed. Interview questions were focussed on the planning of COVID-19 vaccination in their area. Other questions were the problem faced during the implementation of the COVID-19 vaccination program and how it has been tackled.

Results

The factors identified were grouped into three groups: Health system factors, Human resource factors and Community level factors. Health system factors like shortage of vaccines and syringes, tablet paracetamol, online digital method of vaccination registration, overcrowding at the initial stage, and inadequate infrastructure were barriers to vaccination. Fear about vaccine adverse events, even in healthcare workers (HCWs), and overburdened healthcare workers were also factors affecting vaccination. At the community level, high resistance initially and misconception about the vaccine, and also the fear about post-vaccination side effects have an impact on the COVID-19 vaccination program in rural and tribal areas.

Conclusion

The successful vaccination rate among the population needs community leadership and a community-centred approach when conducting outreach and strengthening primary health care in terms of infrastructure, manpower, and capacity building of healthcare staff.

## Introduction

One of the greatest advancements in public health of the 20th century was the development of vaccines to prevent communicable diseases, which have prevented millions of deaths and saved billions of dollars [1]. Willingness to be vaccinated varies among individuals and groups based on various factors, including individual beliefs about vaccine safety and efficacy. Every Food and Drug Administration (FDA)-approved vaccination has been reported to cause reluctance, including delays in or outright rejection of the vaccines by some medical professionals [2,3].

The national policy includes a thorough, secure, and effective immunization effort. Successful vaccine awareness campaigns typically employ multiple strategies [4]. India set a goal to vaccinate 300 million people against COVID-19 by August 2021, the largest immunization program ever. This challenge was announced on January 16, 2021. The homegrown vaccines made by Bharat Biotech, Covishield and Covaxin have been licensed for limited emergency use by the Drug Controller General of India (DCGI) [5]. India simultaneously planned COVID-19 vaccination campaigns in both urban and rural areas. The designated manpower, such as midwives, auxiliary nurses, etc., from all the states, who played a significant part in the healthcare of people in rural India, were trained in COVID-19 immunization. Midwives and auxiliary nurse midwives, who have a far broader reach in the interior and rural areas, were included in the first set of health workers educated in vaccination skills because India planned to have COVID-19 immunization programs in the urban and rural areas at the same time. The government utilized allied healthcare personnel, including pharmacists and public health professionals, to scale up a vaccination campaign. Primary health centres are in charge of effectively implementing the COVID-19 vaccination program in rural areas [6]. So, the study was planned to seek insight into the challenges faced by health personnel in the effective implementation of the COVID-19 vaccination program.

## Materials and methods

A qualitative study was planned to understand the barriers and facilitators of the COVID-19 vaccine implementation program at rural and tribal areas. Ethical approval was obtained from the Institutional Ethics Committee of Government Medical College Akola (MH). The study was conducted in a rural area of Akola district, which lies in the western parts of Maharashtra State and belongs to the Vidarbha region, and is said to be one of the progressive districts in the region. The district has one municipal corporation (Akola Municipal Corporation), six municipalities (Murtizapur; Akot; Balapur; Patur; Telhara and Barshitakli) and 535 Gram panchayat (Total 864 villages). According to the 2011 Census, the district has a total population of 18,13,906, comprising 10,94,165 persons in rural areas and 7,19,741 persons in urban areas accounting for 1.6 percent of the total population of the State [7]. In Akola district, there are 30 primary health centres and 178 subcentres.

The study participants were medical officers from rural and tribal areas who actively planned and implemented COVID-19 vaccination at the primary health centre. A total of 30 medical officers were interviewed. Informed consent was taken from each participant. Each subject gave their informed consent. The Government Medical College (GMC) Akola Institutional Ethics Committee approved the study. Semi-structured interviews were conducted in person between September 2021 and December 2021. All participants were asked regarding problems faced during the implementation of COVID-19 vaccination in their area. Interview questions included: (1) How do you plan for COVID vaccination in your area? (2) What are the problems faced during COVID vaccination? (3) What measures had been taken to solve the problems? (4) What measures will you suggest to implement COVID vaccination in your area successfully? The interviews lasted 20 to 30 minutes and were conducted in the participants' preferred language (Hindi, Marathi, or English). A brief introduction and explanation of the study goals opened the session. Confidentiality was maintained to all participants. Participants were made aware of the existence of no right or wrong responses. Participants were welcomed to express their opinions on the effective implementation and difficulties encountered with the COVID vaccine.

Transcripts of all recordings were created. Two researchers independently read the transcripts before using descriptive words or phrases to code them. Then, areas of interest were assigned to the coded transcripts, and developing themes were described. Review and editing were done on these emergent topics as well as sample transcripts that had been coded. A written presentation of the data is used whenever necessary to highlight study findings with quotes.

## Results

Sample characteristics

A total of 30 medical officers involved in COVID vaccination working at the primary health centres (PHCs) were interviewed. Twelve individuals (12 [40%] women; mean [SD] age, 45.04 [6.9] years) participated (Table 1). Twenty-three (76.7%) participants had work experience of more than five years at PHC in a rural area. A total of 16 (53.3%) medical officers held MBBS degrees.

**Table 1:** Characteristics of study participants

Variable	Number (%)
Age (mean ±SD)	45.04 (6.9)
Male: Female	18:12
Work experience (years)	
< 5 years	07 (23.3%)
> 5 years	23 (76.7%)
Educational qualification	
MBBS	16 (53.3%)
BAMS	14 (46.7%)

Barriers and facilitators in COVID vaccination

The factors identified were grouped into three groups: Health system factors, Human resource factors and Community level factors.

Health system factors

In the initial phase of the mass COVID-19 vaccination program, the barrier to implementation was a shortage of vaccine supply. The basic requirements to conduct an effective vaccination session, like space, manpower, vaccine stock, syringes, tablet paracetamol, etc. were not available initially. One key respondent mentioned that “People unnecessarily argued for the non-availability of vaccine and created a panic situation that was difficult to handle by us. So we took help from the police department.”

Rural or illiterate people did not prefer the online digital method of vaccination registration. Additionally, the network issue for registration was also one of the barriers to vaccination.

“Due to compulsory online registration for vaccination, people keep postponing for it.”

The respondent also mentioned about the inconvenience to the people due to overcrowding highlighting the inadequate infrastructure as a barrier to vaccination.

Human resource factors

Respondent mentioned that initially there was fear about vaccine adverse events even among healthcare workers.

“Initially there was fear about vaccine adverse events even among healthcare workers which led to misconception and rejection for vaccination.”

Most respondents referred to the general shortage of health personnel as a barrier to vaccination. The deficit in human resources affected the vaccination program and overburdened the healthcare workers; it affected non-COVID activities like Universal Immunization Programme, Antenatal check-ups, Field visits, etc. One respondent mentioned that, due to a shortage of manpower in some facilities only one health worker has to perform various tasks in vaccination such as registration, counselling of clients and administration of vaccine.

“There should be proactive involvement of other than health department like for registration, teachers from primary school should be involved. Such involvement should not be on paper but actually on the field.”

The poor attitude among health care workers and lack of commitment due to everyday daily vaccination sessions also were reported to impact negatively on the implementation of the program. Lack of appreciation from higher authority and no additional incentives for staff involved in vaccination was also the barrier observed in the study. Regular training creates awareness among staff. Review from higher authority, especially bureaucrats under the disaster management act, also has an impact on the vaccination program.

Community level factors

Respondents also discussed the attitude of the community towards vaccination. Most respondents viewed the misconception about the vaccine seen to undermine the delivery of COVID vaccine. Respondents stated that there was high resistance initially and misconception about the vaccine particularly. Misconception and superstitious behaviour about the vaccine is present in some communities like Muslims, Buddhist so half of the population of villages is unvaccinated. Healthcare staff also faced high reluctance to vaccination in tribal areas. In Akola district, there were pockets of tribal populations; these populations were also not ready to take vaccines initially due to fear and mistrust.

One respondent said that:

“In a few villages, people were not ready to take the COVID vaccine, only after involving leaders from villages like Sarpanch and house-to-house counselling by Healthcare staff motivated the people for vaccination.”

Misconception and myths about the adverse outcome of the vaccine was a major barrier at the community level. The people were very hesitant and had a phobia about the side effects of vaccination. Pregnant women were also unwilling to take the COVID vaccination due to fear of side effects. One respondent has mentioned that:

“Initially, people were unaware of the safety of vaccines. People had various misbeliefs about the efficacy and safety of vaccine like an increase in death rate after vaccination.”

Some respondents stated that vaccination session timing was not feasible, so people didn’t prefer to lose their daily wages for vaccine.

“In rural areas, in the afternoon, people go to the field, which is more preferred than taking the vaccine, so we can’t cover the total population.”

Post-vaccination side effects like fever, headache, and myalgia created fear among unvaccinated people.

## Discussion

This qualitative study explored barriers and facilitators of the COVID-19 vaccination program in rural and tribal blocks of Akola district. The findings highlighted in our study were classified at three levels: health system, human resource and community. There were several administrative barriers during the initial phase of the COVID pandemic. India is a big country with disparities in health literacy, access to care, and risk perception among the general public by area [7]. The basic requirements to conduct an effective vaccination session like space, manpower, vaccine stock, syringes, tablet paracetamol, etc. were not available initially. In the initial phase, online digital registration for COVID-19 vaccination and internet or mobile network issues in rural areas were critical issues for illiterate people in rural areas. Vaccinating the whole population was challenging even though the country has the largest vaccine-producing units [8]. More than 2.4 billion doses of the vaccine are produced in India each year, in addition to a variety of surgical and medical disposables such as vials, stoppers, syringes, gauze, and alcohol swabs. However, because this necessitates adhering to very strict temperature guidelines, the first barrier was the storage and delivery of the vaccinations until the inoculation sessions [9]. Representatives from the Government of India (GOI) claim that the current healthcare system may not need more personnel to administer the vaccine to healthcare professionals [9].

In relation to WHO standards, India's health worker density continues to be low. In addition to the scarcity, it is extremely difficult to keep skilled health workers in rural and neglected areas [7]. In our study, the general shortage of health personnel and overburdening the healthcare workers affected non-COVID activities like Universal Immunization Programme, Antenatal Care Check-ups, field visits, etc. The frontline HCWs (FLHCWs) were particularly susceptible to increased work stress for a variety of reasons, such as having to work under stressful conditions because of rapidly changing guidelines, the inevitable need to be deployed to new settings and high-risk areas, the lack of adequate technical and human resources, and the worry that they might infect their families after work with COVID-19 [6]. In our study, fear about vaccine adverse events even among healthcare workers (HCWs) was also a reason behind the effective implementation of the COVID vaccination program.

A review by Biswas et al. indicated that 22.51% of 76,471 HCWs worldwide reported COVID-19 vaccination hesitancy. Their main concerns with the vaccine were potential side effects (60%), safety (48%), and how well it works (30%). HCWs in this review also found higher vaccine acceptance in Whites and Asians [10]. A study conducted by Fu et al. on Chinese healthcare workers found that about 50% were unwilling to accept COVID-19 vaccination [11]. However, a study by Saudi healthcare workers Magadmi and Kamel displayed hesitancy toward vaccination [12]. Medical professionals showed a high level of vaccine scepticism, according to Dror et al. [2]. Concerns about the safety of a quickly created vaccine were raised by several respondents who did not follow the recommendations for vaccines. In contrast, individuals who believed they had a higher risk of sickness showed greater vaccine acceptance [2].

The poor attitude among healthcare workers and lack of commitment due to daily vaccination sessions were also reported to impact the program's implementation negatively. Lack of appreciation from higher authority and no additional incentives for staff involved in vaccination was also the barrier observed in the study. Regular training creates awareness among staff. Review from higher authority, especially bureaucrats under the disaster management act, also has an impact on the vaccination program. Rao and Chowdhury also mentioned that frontline workers across the country were the backbone of the Indian public health system [13]. They were underpaid, undervalued, and alienated without any state support or protection. Workloads were raised during the COVID epidemic. The lack of medical insurance, proper governmental support, decreased physical and mental health, and delayed payments all contributed to the financial vulnerability that the healthcare workforce was experiencing [13].

In our study, it was observed that misconception and superstitious behaviour about vaccines were present in some communities like Muslim and Buddhist so half of the population of villages is unvaccinated. Healthcare staff also faced high reluctance to vaccination in tribal areas in the tribal block. The people were very hesitant and had a phobia about the side effects of vaccination [14,15]. Dutta et al. mentioned that various studies have identified myriad factors related to vaccination refusal and hesitancy, including refusal for religious, social, and philosophical reasons, lack of trust in vaccine providers and others, and fear of vaccines and adverse outcomes following immunization [16]. At the same time, King et al. mentioned that mandatory vaccination requirements must be planned with great care; there is no reason to think they are incompatible with human rights law [17]. So there is a need to create awareness regarding vaccination in the community. Vaccination session timing was not feasible for some people, so they didn’t prefer to lose their daily wages for vaccines.

The limitation of our study is that findings cannot be extended to wider area populations with the same degree of certainty as the research findings are not tested statistically. In our study, we focussed on the understanding and explanation of the dynamics of the implementation of COVID vaccination in rural areas, but we haven’t quantified the coverage and issues. This can be better answered with further research with a mixed-method approach.

## Conclusions

In conclusion, our findings were enablers and barriers for COVID-19 vaccination programs in rural and tribal areas. Health system factors like shortage of vaccines and syringes, tablet paracetamol, etc., online digital method of vaccination registration and overcrowding at the initial stage, and inadequate infrastructure were a barrier to vaccination. Fear about vaccine adverse events even in healthcare workers and overburdened healthcare workers were also factors affecting vaccination. At the community level, high resistance initially, misconception about the vaccine, and fear about post-vaccination side effects impact the COVID-19 vaccine program in rural and tribal areas. The successful vaccination rate among the population needs community leadership and a community-centred approach when conducting outreach and strengthening primary health care in terms of infrastructure, manpower, and healthcare staff capacity building.
